# Salivary gland dysfunction markers in type 2 diabetes mellitus patients

**DOI:** 10.4317/jced.52329

**Published:** 2015-10-01

**Authors:** Juan Aitken-Saavedra, Gonzalo Rojas-Alcayaga, Andrea Maturana-Ramírez, Alejandro Escobar-Álvarez, Andrea Cortes-Coloma, Montserrat Reyes-Rojas, Valentina Viera -Sapiain, Claudia Villablanca-Martínez, Irene Morales-Bozo

**Affiliations:** 1 Departamento de Patología y Medicina Oral. Facultad de Odontología. Universidad de Chile; 2 Instituto de Investigación en Ciencias Odontológicas. Facultad de Odontología. Universidad de Chile; 3 Dental student. Facultad de Odontología. Universidad de Chile

## Abstract

**Background:**

Diabetes mellitus (DM) is a chronic disease of the carbohydrate metabolism that, when not rigorously controlled, compromises systemic and organ integrity, thereby causing renal diseases, blindness, neuropathy, arteriosclerosis, infections, and glandular dysfunction, including the salivary glands. The aim of this study was to determine the relationship between the qualitative and quantitative parameters of salivary alteration, which are indicators of salivary gland dysfunction, and the level of metabolic control of type 2 diabetes patients.

**Material and Methods:**

A convenience sample of 74 voluntary patients with type 2 DM was selected, each of whom donated a sample of unstimulated saliva. Salivary parameters such as salivary flow rate, protein concentration, pH, and xerostomia were studied.

**Results:**

There is a positive relationship between the level of metabolic control measured with HbA1 and the protein concentration in saliva (Spearman rho = 0.329 and p = 0.004). The same assay showed an inverse correlation between HbA1 and pH (Spearman rho = -0.225 and *p* = 0.05).

**Conclusions:**

The protein concentration in saliva and, to a lesser extent, the pH may be useful as glandular dysfunction indicators in DM2 patients.

** Key words:**Saliva, type 2 diabetes mellitus, pH, protein concentration, xerostomia.

## Introduction

Diabetes mellitus (DM) is a chronic disease of carbohydrate metabolism and, in its full expression, presents with hyperglycaemia, glycosuria, protein catabolism, ketosis, and acidosis (1). DM is caused by a decrease or absence of insulin secretion or of its biological activity and is associated with specific secondary lesions of the microcirculation as well as with neuropathic diseases and a predisposition to develop arteriosclerosis (2). Type 1 diabetes (DM1) is an autoimmune disease that causes ? cell destruction. Type 2 (DM2), however, affects a higher percentage of diabetic patients. DM2 is influenced by factors such as lifestyle, age, pregnancy, and obesity in addition to a strong genetic component. Eventually, systemic and organ integrity are compromised, causing renal diseases, arteriosclerosis, blindness, neuropathy, infections such as candidiasis, and dysfunction of different glands, including the salivary glands (3).

DM2 requires rigorous self-control from the patient. When such rigor is not achieved, complications linked to the progression of the disease may occur that determine the aggravation of the prognosis and of the quality of life of the patient. The metabolic control of the disease is monitored using the glycated haemoglobin test (HbA1c) (4). This test measures the percentage of haemoglobin attached to glucose in a 90-day period. Levels of HbA1 < 7% are considered compatible with good metabolic control of the disease, while levels > 7% correspond to patients with poor metabolic control and with a higher risk of developing the complications described above (5).

The involvement of the parenchyma of the salivary glands of DM2 patients with modification in its performance and its salivary production and composition has been described (6,7). Saliva has physical-chemical and biological properties that protect the tissues in the oral cavity. Saliva plays helps enable oral physiological processes, such as phonation, mastication, digestion, and food tasting. Considering these aspects, a decrease in salivary production or changes in its qualitative properties may cause a health-related poor quality of life and represent a risk condition to develop oral pathologies such as caries, periodontal disease, angular cheilitis, and candidiasis (8-10).

Salivary gland damage in DM2 patients can cause qualitative and quantitative changes in the saliva. Xerostomia (dry mouth sensation) is also more frequent in these patients than in non-diabetic patients.

Among the altered salivary parameters described in DM2 patients is the salivary flow rate (SFR), the pH, the salivary protein concentration and the xerostomia. Any of these parameters, singly or jointly, may reflect salivary gland damage (11).

It is of great interest to identify the changes in the salivary parameters that suggest glandular deterioration in DM2 patients according to the level of metabolic control of the disease, as adequate maintenance of the blood HbA1 levels may be more important in preventing tissue damage than the disease itself.

To our knowledge, there is inadequate evidence on the association of salivary parameters with the levels of the HbA1 test in the same patient, but such information would help to determine the susceptibility to presenting oral diseases linked to these parameters and aid in both evaluating glandular damage and improving the therapeutic approach.

The aim of this study is to determine the possible association between the alterations of the qualitative and quantitative salivary parameters that indicate glandular dysfunction and the metabolic control levels in patients with type 2 diabetes mellitus.

## Material and Methods

-Sample

A convenience sample of 74 voluntary subjects was selected from the Chilean Association of Diabetics (ADICH, for its acronym in Spanish). The selection was based on a non-probabilistic intentional method or done by convenience sampling, given the essential research requirements (inclusion and exclusion criteria) with which the units of analysis had to comply. This study was performed on the premises of the ADICH, following the universal bioethics principles expressed by the Helsinki Declaration (12). Each subject had to sign a form indicating informed consent, previously approved by the Ethics Committee of the Faculty of Dentistry of the University of Chile.

-Selection Criteria 

•Inclusion

Subjects of either sex over 30 years of age with a confirmed diagnosis of type 2 diabetes by the ADICH following the established criteria of the Ministry of Health (MINSAL, for its acronym in Spanish) (1).

•Exclusion

Subjects with rheumatologic conditions, with previous head and neck radiotherapy, terminal diseases with neurological damage, acute inflammatory oral conditions, or pregnancy were excluded from this study.

-Determination of unstimulated salivary flow rate (SFR)

All saliva samples were taken by a single dental surgeon. Following a distilled water mouthwash and 5 minutes under relaxed conditions (13) and deposit saliva for 5 minutes in a previously labelled sterile tube as described by Navazesh *et al.* (14). The tube was later weighed by gravimetry, assigning a specific weight of 1.005 g/ml to the fluid, and the calculated total volume was expressed in ml/min.

-Saliva pH measurement 

The pH of the saliva samples from each individual was determined using saliva from the same tube for which the SFR was measured. A digital pH meter (PL-600 EZDO-OMEGA model following the ISO-9001 regulation) automatically provided the pH value digitally to 2 decimal places (15).

-Xerostomia evaluation

To evaluate the dry mouth sensation, the validated Fox test was applied (16) that consist in evaluate the patient’s subjective complaints that may include oral dryness, difficulty swallowing, problems speaking without additional liquid, burning sensation in the mouth, sensitivity to acidic or spicy foods and taste changes.

-Protein concentration in saliva

Kit BioRad protein assay (BRL SD 500-0002, Richmond CA, USA) and standards bovine albumin (BSA, Sigma) were used. Saliva samples analyzed were processed in duplicate, in serial dilutions of 1: 100 to 1: 200 and absorbance at 595 nm was determined. With the data obtained, a calibration curve was plotted and the concentration of total protein in mg / mL was estimated. A one-dimensional electrophoretic analysis. Aliquots of whole saliva containing equal amount of proteins were subjected to separation on SDS-polyacrylamide (10%) under reducing conditions according to the method described by Laemmli *et al.* (17) and developed by staining with Coomassie Blue. Polypeptide bands that will be stained with purple dye were identified. The molecular weight was determined apparent of these bands, defined against calibration standard protein gels (Precision Plus Protein Standards, Dual Color, Bio Rad Catalog # 161-0374). The unit of measurement of the concentration of proteins in saliva was ug / mL.

-HbA1c

HbA1c blood was used as the gold standard. This test is performed routinely in the ADICH to establish metabolic control of the disease using the Variant II Bio Rad brand equipment, certified to the National Glycohemoglobin Standardization Program of the United States (18). The percentage describing the methodology, is obtained by affinity chromatography calculates the total and hemoglobin HbA1c indirectly through a linear regression equation and the immunoassay, which specifically determines the percentage of HbA1c by reacting monoclonal antibodies. It was considered according to the protocol described by the committee of diagnosis and classification of diabetes mellitus (19), a higher percentage of HbA1c to 7%, metabolic descompensation, while values below this percentage, compensation was considered metabolically.

-Statistical Analysis

A descriptive statistical analysis was performed based on the average and standard deviation for SFR, pH, and protein concentration. Normality for the quantitative variables was evaluated based on the Shapiro-Wilk test. For the quantitative variables, the values were calculated for a 95% confidence interval. Student’s t-test was used to compare the measurements between patients with DM 2 and HbA1 > 7% and patients with DM2 and HbA1 < 7%. To establish correlations between HbA1 and the variables SFR, pH and protein concentration, the Spearman correlation coefficient was used. Statistical differences with a level of significance under 0.05 (*p* < 0.05) were accepted. The Stata 1.1 software was used to perform the statistical analysis.

## Results

Of the subjects, 53 were women (71.6%) and 21 men (28.4%). The mean age for the total sample was 62.13 (SD 10.13) years, 63.3 (SD 9.23) years for men and 59.3 (SD 11.7) years for women. Twenty-six subjects had levels of HbA1 under 7%, while 48 subjects had levels of HbA1 over 7%. An average of HbA1 level of 8.63 (SD 2.27) was determined for the total sample. The minimum value was 5.7%, and the maximum was 13.7%.

 Table 1 shows the disaggregated values for the salivary parameter according to the metabolic control of DM2 (HbA1 < 7% and HbA1 > 7%).

**Table 1:**
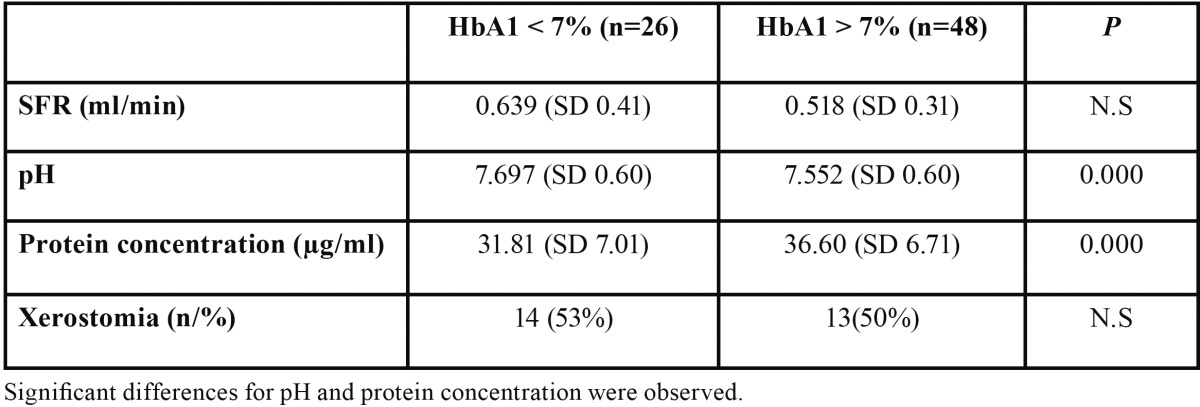
Comparison of salivary parameters for DM2 patients with HbA1 levels over and under 7%.

The average SFR value for the total group was 0.561 (SD 0.354) ml/min.

The average of the saliva pH values for the total group was 7.60 (SD 0.604). Significant differences were found between the two groups (t-test *p* = 0.000).

The average protein concentration in saliva for the total group was 34.92 (SD 7.152). Significant differences were found between the two groups (t-test *p*=0.000).

Of the patients with DM2 and HbA1 < 7%, 14 (53%) referred xerostomia, while 13 (50%) of the patients with DM2 and HbA1 > 7% referred this symptom.

**Figure 1:**
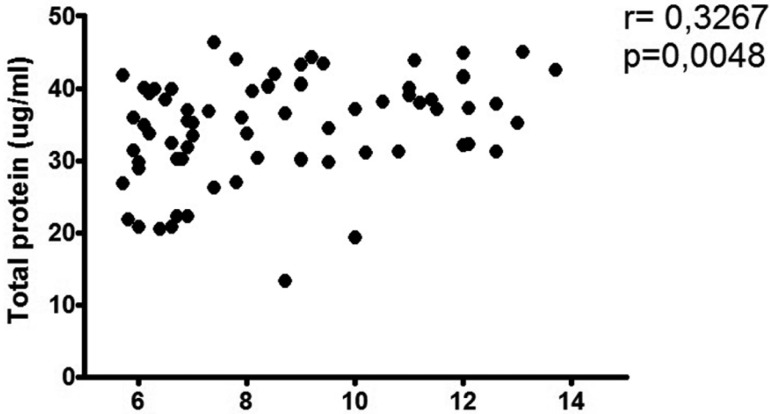
Association between protein concentration in saliva and the levels of glycated haemoglobin (HbA1c). The levels of protein concentration in saliva were determined using spectrophotometry. Positive association between the two variables is observed.

As shown in figure 1, the Spearman correlation coefficient showed a positive association between the level of metabolic control measured by HbA1 and the protein concentration (Spearman rho = 0.329 and *p* = 0.004). The same test also showed an inverse correlation between HbA1 and pH (Spearman rho = -0.225 and *p* = 0.05). SFR and HbA1 showed no association.

## Discussion

The aim of this study was to evaluate salivary gland dysfunction in patients with DM2, measuring the different levels of metabolic control with the HbA1 test and examining the SFR, pH, protein concentration and xerostomia parameters.

Although the DM2 patients with HbA1 > 7% showed a lower salivary flow than the DM2 patients with HbA1 < 7%, this difference was not statistically significant when comparing the two groups. A correlation between the SFR and HbA1 variables was not observed, which would suggest a lower salivary flow linked to the worsening of the metabolic control of DM2. While there is scarce evidence to link the SFR with HbA1, several studies suggest that patients with diabetes mellitus undergo glandular damage that may translate into a lower salivary flow than in healthy subjects. Some authors (4,11) consider that this decrease is related to an increased diuresis or polyuria, involving a decrease in extracellular fluid and consequently in saliva production. Other authors explain this as a consequence of dehydration from glycosuria that would be more evident in cases of metabolic decompensation (7). However, this result was not observed in our study. Furthermore, according to our results, independently of the metabolic control of the disease, the salivary flow levels of DM2 patients are above the hyposalivation range (SFR < 0.2 ml/min), a risk category for the onset and progression of oral cavity diseases such as periodontitis and caries (9,10).

Our study found a higher protein concentration in saliva for DM2 patients with HbA1 > 7% compared to DM2 patients with HbA1 < 7%. In addition, a positive association was observed between the degree of metabolic decompensation and the protein concentration in saliva (Spearman rho = 0.329 and *p* = 0.004). The increase in protein concentration in saliva is probably due to the reduced salivary fluid secretion described in diabetics (20). Another explanation for an increased presence of saliva proteins in DM2 subjects would be oral inflammatory conditions such as periodontitis (21). In this case, the origin of the proteins in the total saliva composition would be mainly from the crevicular fluid. Although the periodontal health status was not evaluated in our study, it is possible that an increase in the severity of periodontitis in decompensated DM2 patients might influence the protein composition parameter, as described in the literature (9,10,21). Although there are several explanations for the higher protein concentration phenomenon in saliva in DM2 subjects, we suggest that more information on its origin, characterisation, and individualised information is required.

A lower pH in subjects with diabetes than in healthy subjects has been reported in the literature (4). However, there is insufficient evidence correlating saliva pH with HbA1 values, as performed in our study. According to our results, there was a low but inverse correlation between the variables (Spearman’s rho = -0.225 and *p* = 0.05). That is, the pH becomes more acidic as the diabetic decompensation increases. These changes in pH have been attributed in some studies to the decrease in bicarbonate in diabetic patients (22), which could be more evident as the disease progresses. Although a drop in the pH may be a risk factor for oral cavity disease such as periodontitis or caries (23), the values detected in the participants of our study, independent of the degree of metabolic control, continue to be considered normal or neutral and of low risk in relation to the etiopathogenesis of these oral pathologies.

Regarding xerostomia, diabetic patients are more subject to present this condition than the non-diabetic population, as described in the literature (24,25). There is insufficient evidence to associate this symptom with HbA1. In our study, the differences between patients with good and poor metabolic control of DM2 and the presentation of xerostomia were not observed. We also found no correlation between xerostomia and the measured parameters (SFR, pH and protein concentration in saliva). Furthermore, although 58% of our sample had hypertension and were under drug treatments that have been associated with dry mouth sensation (26), we did not observe an association with xerostomia (data not shown). Thus, we suggest that although a dry mouth sensation may be associated with diabetes, it is not a valid indicator of glandular dysfunction. Other salivary qualitative parameters were not analysed in this study, and thus we suggest, in accordance with literature, that xerostomia could be a result of multiple factors, including the psychological status of patients with this condition (27).

Our results show for the first time that decompensation, as measured by HbA1, may be linked to altered salivary parameters. According to our study, the protein concentration in saliva and, to a lesser extent, the pH may be useful as glandular dysfunction indicators in DM2 patients. However, for this purpose, we believe that broadening our sample would make our results more reliable but also that complementing our results with other studies including different salivary parameters would, jointly or individually, be useful in monitoring DM2 by offering a less invasive and lower cost alternative for the metabolic control of type 2 diabetes mellitus.
